# Epidemiological study of suicide by physical methods between 1993 and 2013 in Ilam province, Iran

**DOI:** 10.1186/s12888-017-1461-5

**Published:** 2017-08-23

**Authors:** Yosra Azizpour, Kourosh Sayehmiri, Khairollah Asadollahi, Satar Kaikhavani, Maryam Bagheri

**Affiliations:** 1 Department of Clinical Epidemiology, Faculty of Health, Ilam University of Medical Sciences, Ilam, Iran; 2 Psychosocial Injuries Research Center, Ilam University of Medical Sciences, Ilam, Iran; 3 Clinical Epidemiology, Department of Social Medicine, Faculty of Medicine, Ilam University of Medical Sciences, Ilam, Iran; 4 Department of Clinical Psychology, Faculty of Medicine, Ilam University of Medical Sciences, Ilam, Iran

**Keywords:** Epidemiology, Invasive, Physical method, Suicide, Ilam, Iran

## Abstract

**Background:**

Suicide by aggressive physical methods such as firearms, hanging, and jumping is well known; however, different factors may influence a person while selecting a particular method. The aim of this study was to investigate the epidemiological factors involved in the selection and use of different physical methods for suicide over a long-term period in Ilam province, Iran.

**Methods:**

The present study was conducted retrospectively between 1993 and 2013 using recorded data from a comprehensive system for registration of suicide attempts in Ilam University of Medical Sciences. The epidemiological characteristics included person, time and place variables, and the outcomes of the suicide attempts. The chi square, univariate and multivariate logistic regression models were used for data analysis.

**Results:**

Totally, 1516 suicide attempts were evaluated (the annual incidence rate: 19/100,000 individuals). The most commonly used suicide method in females (88.4%) and males (38.9%) was self-immolation. Furthermore, the annual incidence rate among males and females was within the age group of 15–24 years (24.6 and 47.8/100,000 individuals). The risk of death by suicide in the age group of 55–64 years was 2.93 compared with the age group of 10–14 years (OR = 2.93; 95% CI = 0.64–13.54, *P* = 0.168).

**Conclusion:**

This study revealed that self-immolation was the most selected physical method of suicide and had the highest incidence rate, and inflicted the survivors with severe physical and mental complications. In order to reduce the use of physical methods, especially self-immolation, life skills training becomes more important than ever.

## Background

Completed suicide is defined as the death that resulted from an intentional attempt to take one’s own life, but attempted suicide is the act of self-injury from a decision to end one’s own life, but does not result in death [1]. The methods used for committing suicide are divided into two categories; physical and chemical. Physical methods include jumping from a height, motor vehicle injuries, suicide on train railways, self-immolation, drowning, firearm application, cutting with sharp instruments, choking and hanging [2]. The leading suicide method varies considerably among different societies. Men worldwide, often use violent methods [3]. Completed suicides via firearm (60.6%) and hanging (20.4%) were ranked first and second among men in the United States of America (USA); however, ranked the first (35.7%) and third (16.9%) among women respectively [3]. In addition, hanging (52.4%) and firearm (22.1%) were the first and second methods of suicide among males in Brazil; however, hanging (37.6%) was the first among females in Brazil [3]. Notably, hanging is the first suicide method among men and women in the age group of 15–24 years in the European countries (the annual incidence rate: 5.5 /100,000 and 1.1 /100,000 people respectively) [4]. Hanging was a common suicide method in China, Hong Kong, Japan, South Korea and Malaysia (one of the predominant methods of suicide in Asia); however, firearm was the second and third method of suicide in Philippines, Pakistan, Saudi Arabia and Turkey [5]. Self-immolation was rarely attempted in the West [6], but it was used as the second and third common method in India [7–9]. Remarkably, self-immolation was the third common method in Iran [10]. However, suicide behaviors and suicide attempts are forbidden in Islam based on religious beliefs [11].

There are 31 provinces in Iran and Ilam as the smallest one, is placed in the west of the country bordering Iraq. Ilam city is the provincial center and the population of the province is 557,599 based on the National Population and Housing Census in 2011. Ilam province shares its borders with three Iranian provinces and Iraq in the west with a common border of 420 km. The residents of the province experienced sever bombing during the war (Iran-Iraq; 1980–1988) and 2382 people were killed, which may be one of the main reason for the highest incidence rate of suicide and psychological problems in the province compared with the whole country. Durable war may lead to post traumatic stress disorders (PTSD), which can increase the risk of suicide attempt following psychological pain. There is evidence of a relationship between suicide thoughts and PTSD among Kurds in the west of Iran [12].

Based on a report by world health organization (WHO), some parameters are crucial for an effective suicide prevention program in a society. The basic step is enhancing the availability and validity of the data registration system from health centers and hospitals. Much evidence supports the idea that easy access to the means of suicide increases the risk of suicide. Accordingly, suicide can be prevented by restricting access to the means, which may differ between locations and cultures. In addition, researchers proposed that biological, psychological, sociocultural and economic factors all have a role in suicidal behavior. Therefore, identifying the risk factors specific to high risk regions or vulnerable individuals is an important strategy in suicide prevention programs in many societies. Preparing a comprehensive suicide prevention program requires a close cooperation between different government sectors. The health minister can take the responsibility to manage and coordinate different activities with other ministries. A helpful suicide prevention program can be accessible through this multispectral collaborative effort. According to researches by WHO, the followings can be considered as suicide prevention objectives: developing the related researches in order to identify the vulnerable individuals, groups and geographical regions; developing epidemiological research to identify biological, psychological, cultural and environmental risk factors in suicide; revising the rules that give access to means of suicide; improving social awareness regarding suicide risk factors, vulnerable groups, and supportive centers for suicide behavior besides the protective factors; holding educative programs and seminars regarding suicide behaviors; improving public education to decrease or eliminate the stigma related to suicidal behaviors and psychosocial problems; and revising the policies for health problems and priorities regarding health problems [13].

Evaluating different aspects of a problem is the most important step in problem solving. Accordingly, in suicidal behavior (violent and nonviolent), as one of the important social problem, it is unavoidable to analyze the personality, different causes of suicidal behaviors, means of suicide, or physiological and geographical changes. On the other hand, the pattern of suicide is different for both genders in either violent or nonviolent methods. Obviously, a deep understanding and evaluation of the different aspect of suicidal behaviors may present a new horizon to psychologists and psychotherapists in order to organize the best prevention program for suicide.

There is lack of comprehensive information about the distribution of physical suicide methods according to age, marital status, level of education, employment status, place of living, season, motivation of suicide, housing status, committing suicide status, ethnicity and time of suicide in males and females in Ilam province. In addition, the effect of these important factors on the likelihood of complete suicides by physical methods was not clear; therefore, this study was launched to evaluate these queries.

## Methods

By a retrospective study, all records of suicide attempts committed by physical methods between April 1993 and March 2013 in Ilam province were evaluated.

The information was obtained from the comprehensive system for suicide registration in Ilam University of Medical Sciences.

A suicide committee was established by the Psychosocial Injury Research center at Ilam University of Medical Sciences, and all cases of completed and attempted suicides are recorded by an online program, known as Farabar. Data is transferred from different medical centers monthly to a basic center of the suicide committee (suicide registration program, Farabar). The suicide committee functioning as a bank of suicide cases or suicide attempts information reservoir, can provide this information for researchers. Complete information from different medical centers should be recorded monthly in Farabar and transferred to the main documentation center of the suicide committee. Since different medical centers may contribute to medical procedures, having repeated records of a patient is logical. Consequently, all data are evaluated by the experts of the suicide committee in order to delete the repeated ones before final saving. It is noticeable that information must be kept secure during studies. Researchers use information according to specific variables including age, means of suicide, sex, time, etc.

Regarding data gathering, it should be mentioned that the victims of attempted suicides either go directly to a hospital’s emergency room themselves or are referred to an ER after visiting a health center, clinic or doctors’ office. Then, a practitioner starts therapeutic approaches as soon as possible. The reason for suicide is noted based on the testimony of the patient or the persons who accompanied her/ him, and soon after, the police should be informed to do legal documentation and registration. Finally, a nurse inserts the data into the Farabar system. In the case of a completed suicide, a forensic examination is performed in the hospital. The exact cause of death is clarified and a death certificate is signed. A nurse would add the method of suicide to the Farabar system after Forensic specialist assessment. Sometimes, patients are referred to legal medical centers directly and data are collected there. In Iran, a formal forensic medical letter is mandatory for burial permit. So, no case of completed suicide is missed.

Suicide attempts lead to two different statuses; feeling of embarrassment and repeated suicide. However, if the family is sympathetic towards the patient, rather than ignoring him/her, it may help the patient to improve the condition [14]. Regarding the burial ceremony of suicide victims, although suicide is inhibited in Islam, the burial ceremony is held similar to other people.

Researchers used the recorded information according to the epidemiological characteristics including personal variables: gender, age, education level, marital status, employment status, motivation of suicide (Psychiatric problem was defined as mood disorders including depressive disorders, bipolar disorders, anxiety disorders and schizophrenia disorders); housing status (Organizational houses: Non-private accommodations as homes/apartments that are offered by ministers or governmental institutions to their employees. Gratuitous houses: The government prepares free accommodation for homeless people which is called gratuitous accommodation); ethnicity (People speak different languages such as Kurdish, Lori, Arabic and laki in Ilam province. Accordingly, they are traditionally allocated to different ethnicity groups); family structure (A nuclear family consists of parents and their child/ children. An extended family includes parents and their child/ children and other family members such as grandmother, father, uncle, aunt, etc.); the status of committing suicide; place variables (place of living); time variables (season, month and time of the day (Morning (midnight until 11:59AM), afternoon (12 pm–3.59 pm), evening (4 pm–7 pm), night (7 pm–12 pm)) and suicide method outcomes (death or survival). It should be noted that different physical methods including hanging, firearms, self-immolation, use of sharp tools, as well as other methods such as jumping from height, electric eclipse, air injection and drowning were considered. In order to estimate the specific suicide incidence rate in 100,000 individuals, the population census of Ilam province in 2011 was considered; the population at risk was >10 years and was calculated based on age group, gender, place of living, marital status, education level and employment status. The ethical committee of Ilam University of Medical Sciences approved the study.

### Statistical analysis

Frequency, percentage and incidence rate were used for data description. Association between nominal, ordinal and categorical variables was considered using the chi-square test. In order to find the association between death hazard after suicide and other covariates, univariate and multivariate logistic regression (using Forward likelihood method) models were used. Firstly, different variables were inserted into the univariate logistic regression separately. Then those with significance level *(P < 0.2)* were inserted into the multivariate logistic regression. The covariate with *P* < 0.05 was excluded from the final model. *P ≤ 0.05* was regarded as a significant level.

Unfortunately, suicide information was not recorded in the registration system for 2000 and 2002. In addition, the information was incomplete in 2007 and 2010. The missing data resulted from the administrative changes in the health care system during those years. It should be mentioned that we secluded missing data without estimating for it.

## Results

The distributions of both physical and chemical suicide methods in Ilam province from 1993 to 2013 were: self-poisoning (79.1%), self-immolation (12.5%), hanging (2.5), use of sharp tools (2%), firearm (0.7%), other physical suicide methods including electric eclipse, jumping from height, drowning and use of air injection (0.2%), and unknown methods (3.1%).

**Fig. 1:**
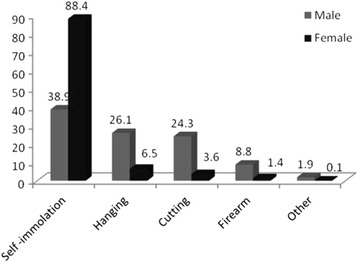
The relative frequency of different methods of physical suicide in males and females

Overall, 1516 cases of physical suicide were identified in this study and entered into analysis (the annual incidence rate: 19 /100,000 people) including 571 males (37.7%) and 945 females (62.3%). The most commonly used suicide method in females (88.4%) and in males (38.9%) was self-immolation (Fig. 1).

The annual incidence rates of completed and attempted suicides via physical methods in females were 15.11 and 8.67 /100,000 respectively, while in males were 6.59 and 7.38 /100,000 respectively. However, final outcomes of 6 cases of physical suicides were unclear.

The highest annual incidence rate of suicide by physical methods in males and females was observed within the age group of 15–24 years. Furthermore, the annual incidence rate of suicide in males and females was higher among singles, middle school degree, unemployed people and those living in rural areas. There was a significant relationship between suicide (in male and female) and age (*P* = 0.023), marital status (*P* = 0.0001), educational level (*P* = 0.0001), employment status (*P* = 0.0001) and location (*P* = 0.004) (Table 1).

**Table 1:** The incidence rate of suicide by physical methods based on demographic variables during 1993–2013 in Ilam province

Variables		Male		Female		*p*-value
		No. (%)	Annual incidence rate	No. (%)	Annual incidence rate	
Age	10-14 years	15 (2.6)	4.35	40 (4.2)	12.36	0.023
	15-24 year	273 (47.8)	24.6	517 (54.7)	47.8	
	25-34 years	173 (30.3)	17.04	223 (23.6)	21.81	
	35-44 years	47 (8.2)	6.97	74 (7.8)	11.14	
	45-54 years	27 (4.7)	6.71	41 (4.3)	10.25	
	55-64 years	22 (3.9)	8.47	23 (2.4)	8.34	
	≥65 years	14 (2.5)	5.73	27 (2.9)	13.37	
Marital status	Single	391 (68.6)	20.89	455 (48.3)	29.48	0.0001
	Married	177 (31.1)	8.29	470 (49.8)	22.09	
	Separated and widow	2 (.0.4)	4.93	18 (1.9)	6.06	
Level of education	No elementary	45 (8.5)	8.66	187 (22.4)	21.14	0.0001
	Elementary school	83 (15.7)	14.60	194 (23.3)	28.87	
	Middle school	143 (27)	20.78	181 (21.7)	36.24	
	High school	216 (40.8)	16.69	236 (28.3)	22.52	
	Academic	42 (7.9)	4.78	35 (4.2)	4.41	
Employment status	Employed^a^	175 (34.7)	8.38	33 (3.8)	8.29	0.0001
	Unemployed	251 (49.8)	49.03	242 (28.1)	109.91	
	Housewife	0 (0)	0	430 (49.9)	20.55	
	Student	78 (15.5)	8.91	156 (18.1)	17.88	
Place of living	Urban	246 (56.6)	9.52	355 (48)	13.92	0.004
	Rural	189 (43.4)	12.98	385 (52)	27.30	

^a^Including: Farmers, workers, office workers and self-employed

The frequency of suicide attempts was higher in males during spring (26.4%) and in females during summer (26.2%). Furthermore, the highest frequency of suicide in males and females occurred in April (11.4%) and August (10.4%) respectively. Regarding the motivation of suicide, family conflict and psychological problems were the most frequent causes of suicide in both males and females (Table 2).

**Table 2:** The frequency distribution of suicide by physical methods based on other variables during 1993–2013 in Ilam province

Variables		Male	Female	Total	*p*-value
		No. (%)	No. (%)	No. (%)	
Season	Spring	151 (26.4)	243 (25.7)	394 (26)	0.670
	Summer	136 (23.8)	248 (26.2)	384 (25.3)	
	Autumn	146 (25.6)	222 (23.5)	368 (24.3)	
	Winter	138 (24.2)	232 (24.6)	370 (24.4)	
Motive of suicide	Psychic problem	161 (37.3)	226 (30.7)	387 (33.1)	0.0001
	Physical problem	3 (0.7)	8 (1.1)	11 (0.9)	
	Economical problem	16 (3.7)	14 (1.9)	30 (2.6)	
	Family conflict	203 (47)	468 (63.5)	671 (57.4)	
	Educational problem	6 (1.4)	14 (1.9)	20 (1.7)	
	Addiction problem	29 (6.7)	1 (0.1)	30 (2.6)	
	Unemployed problem	14 (3.2)	6 (0.8)	20 (1.7)	
Housing status	Personal	172 (36.4)	216 (25.1)	388 (29.1)	0.0001
	Organizational	6 (1.3)	12 (1.4)	17 (1.4)	
	Gratuitous	254 (53.8)	533 (61.9)	787 (59)	
	Rental	40 (8.5)	100 (11.6)	140 (10.5)	
Family structure	Nuclear family	353 (93.6)	636 (80.8)	989 (85)	0.0001
	Extended family	24 (6.4)	151 (19.2)	175 (15)	
Status of committing suicide	Alone	355 (93.9)	728 (92)	1083 (92.6)	0.249
	At the presence of others	23 (6.1)	63 (8)	86 (7.4)	
Ethnicity	Lor	32 (8.4)	54 (6.8)	86 (7.3)	0.576
	Kord	344 (90.5)	734 (92.3)	1078 (91.7)	
	Others	4 (1.1)	7 (0.9)	11 (0.9)	
Day Time of suicide	Morning	44 (18.8)	151 (24.1)	195 (22.6)	0.048
	Afternoon	33 (14.1)	117 (18.7)	150 (17.4)	
	Evening	121 (51.7)	262 (41.8)	383 (44.5)	
	Night	36 (15.4)	97 (15.5)	133 (15.4)	

**Fig. 2:**
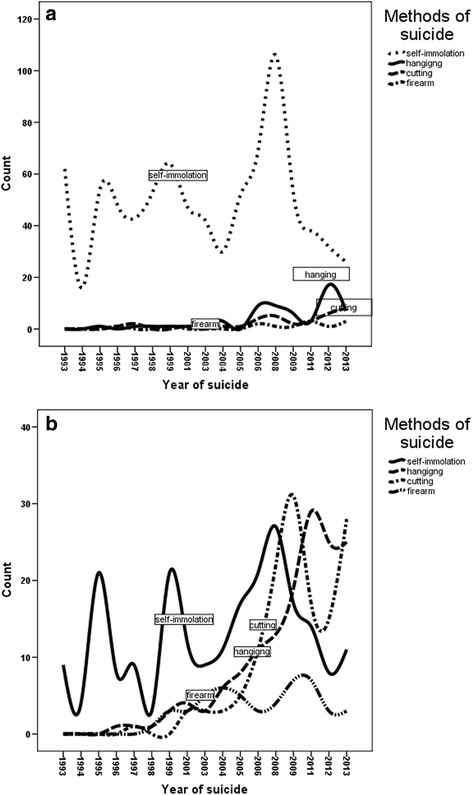
The trend of suicide by physical methods in (**a**) females and (**b**) males during 1993–2013 in Ilam province

The findings of this study showed that the risk of death in the age group of 55–64 years was 2.93 times more than in the age group of 10–14 years insignificantly (OR = 2.93; 95% CI = 0.64–13.54, *P* = 0.168) (Table 3). The results also showed that females committed suicide more frequently by self-immolation; however, suicide was committed more frequently in males by hanging, firearms and sharp instruments (Fig. 2a and b).

**Table 3:** The Prediction of completed suicide by physical methods using multivariate logistic regression model during 1993–2013 in Ilam province

Variables		Adjusted Odds ratio	(95% CI)	*p*-value
Age	10-14 years	1.0^a^		
	15-24 year	0.54	0.20–1.34	0.182
	25-34 years	0.50	0.20–1.29	0.152
	35-44 years	0.83	0.29–2.37	0.730
	45-54 years	1.03	0.33–3.24	0.957
	55-64 years	2.93	0.64–13.54	0.168
	≥65 years	1.43	0.37–5.51	0.606
Gender	Male	1.0^a^		
	Female	1.87	1.35–2.60	0.0001
Family structure	Nuclear family	1.0^a^		
	Extended family	2.00	1.22–3.26	0.006
Ethnicity	Lor	1.0^a^		
	Kord	2.34	1.27–4.30	0.006
	Others	2.94	0.48–18.04	0.245

^a^Reference

## Discussion

### Gender and suicide

The results of the present study revealed that self-immolation was the most frequent suicide method among women in Ilam province. Self-immolation was also a common method for suicide in India, Pakistan and Sri Lanka but was less frequent in the western countries [5]. The significant difference between our findings and the information from other countries may be due to social, cultural and behavioral factors, as well as the rate of accessibility to means of suicide. However, there may be some unknown factors that play important roles in suicide behavior and selecting a certain method for suicide.

Based on the Iranian culture and relevant rules, men can ask for marriage separation more easily than women. Usually, for women, suicide attempts are more acceptable than marriage separation in some domestic regions [15]. Nowadays, women and girls are aware of their personal and social rights more than ever; however, in some parts of Iran such as Ilam, women’s knowledge about their rights is limited [14]. Consequently, they become frustrated and vulnerable to social injuries. Another social problem is the relationship between boys and girls, which is unacceptable among most Iranian families [14]. Women may have idealistic thoughts and might experience different kinds of social and cultural conflicts through mass media, which may lead to introversion and apathy [16]. It seems that changes in the life styles and lack of cultural, educational, social and economic facilities, for example in rural areas compared with the urban areas, may result in higher incidence rate of suicide. In addition, the impact of war and its different consequences should not be forgotten and Ilam as one of the provinces with a long border with Iraq was more affected. Most of the residents of this province experienced different psychosocial, economical and physical injuries during the 8 years’ war between Iran and Iraq [17]. Ilam province is a religious society; however, it seems suicide attempters do not obey Islamic rules regarding suicide.

Ryazi et al. focused on the socio-cultural and psychological factors of suicide in females living in Ilam (Iran) and Dushanbe (Tajikistan). Home violence, cultural use of force (threatening to attempt self-immolation, as a common phrase among parents), forced marriage, the lack of privacy at home, irrational fanaticism of honor, polygyny and too much responsibilities were reported as the most common causes of suicide in females [18]. A study by Kashi et al. reported that Lorestan province, which has different cultures and values in city, village and nomadic societies, was one of the underdeveloped regions in the country with cultural, social and economic poverty. Much evidence supports the relationship of these areas of poverty with social problems [19]. The most important causes of suicide among female living in Lorestan province were swear words and false accusations by males, spouse joblessness, spouse addiction and violence and marital conflicts. Furthermore, when there are serious problems with the spouse’s family, divorce is an uncommon option and a taboo [20]. Lorestan is located at the east of Ilam province. It has a similar culture with Ilam province and geographical factors that may affect peoples’ life and behaviors including suicide attempts. Zebardast and Roosta studied the side effects of regional disparities between Hamedan and Markazi provinces. They showed that cultural characteristics, demographic characteristics, health conditions and educational status were more developed in Markazi province than Hamedan [21]. The results of another study in Hamedan showed that depression, social dysfunction, physical disorders, insomnia and anxiety was reported mostly in women who experienced self-immolation [22]. However, forced marriage, lack of privacy, lack of private accommodation (living with spouse family), and cultural acceptability of self-immolation in the society were considered as the main causes of female self-immolation in Kermanshah [23]. Overall, negative cultural believes besides the cultural and economic poverty resulted in the high occurrence of female suicide in underdeveloped regions.

Women committed self-immolation more than other methods since it has a cultural acceptability. Women usually threaten to self-immolate themselves during a family conflict in order to change the worse conditions, to get more gain as an escape route from unsolvable problems or from a genuine desire to die. Modeling from other cases of self-immolation, self-immolation repetition [23] and the availability of self-immolation aid such as oil may predispose people to attempt this method [24].

### Age and suicide

The results of this study showed a higher incidence rate of suicide by physical methods in males and females aged 15–24 years old. This finding was similar to a study on developing countries that reported a higher incidence rate among people under 30 years old [25]. Integrated analysis of suicide in Iran showed that the majority of suicide cases were carried out by the young population [10]. Children, especially the girls, are dependent on their families until marriage in almost all cities of Iran, particularly in the smaller cities such as Ilam. On the other hand, the generation gap between parents and their children may lead to comprehensive changes in the social values [26], and these factors may result in family problems, misunderstandings and conflicts. The high prevalence of unemployment among the youths, especially for the educated people, can cause disappointment and frustration [27]. Based on a study by Bazrafshan et al., family loss, parental divorce, parent-child and sibling friction, romantic issues, family history of drug abuse, socioeconomic status, educational issues and psychiatric disorders such as depression were the reasons for suicides among Iranian adolescents [28]. The elderly people receive much care and respect based on the Iranian religious and cultural background, which [29] may be a good reason for the reduced rate of suicide in this group.

### Marital, occupational, educational status and suicide

Based on demographic variables in our study, the single individuals, the unemployed people and the individuals with middle education had the higher incidence of suicide by physical methods. According to a study that was done in India, married female individuals and individuals with middle education had a higher incidence rate of suicide by hanging [30]. Incidence of completed suicides, caused by jumping from height, was more common in married individuals in Hong Kong [31]. The highest frequency of self-immolation in Pakistan occurred in married people, housewives and illiterate individuals [32]. This discrepancy can originate from socio-economic conditions as well as the customs and cultural characteristics of the society compared with Iran’s society.

### Season and time of suicide

Season is another important factor affecting the suicide behaviors. According to the results of the present study, the highest percentage of suicides by physical methods happened during spring, at evening among males and during summer, at evening among females. However, the difference between seasons and suicide in male and female was not significant in the current study.

A study from the South [33] and East [34] parts of Iran reported that self-immolation happened mostly in winter and summer respectively. Similar to our findings among males, a concluded study showed that hanging reached a peak level in spring among males and females [35]. In another study carried out in Italy, violent suicides in males had a peak level in spring [36]. It is plausible that the sun exposure leads to fluctuations in serotonin system and serotonin-related behaviors [37]. It seems that the time of suicide attempts can vary in accordance with the geographical locations, weather conditions, biological and psycho-emotional factors.

### Logistic regression

Logistic regression analysis showed that the risk of death following suicide in the age group of 55–64 years was 3 times more than the age group of 10–14 years. It seems that people in the former age group are not strong enough to cope with the psychological and physical stresses/ damages. Since they may lose their hope of the future, they may select a lethal suicide method. However, it seems that suicides among youths are with an intention to attract attention rather than from a real decision to die. The risk of death in women was two times higher than in men. The most common method of physical suicide is self-immolation, which is more common in women and leads to an increased risk of death in women.

### Violent suicide in Iran

Some of our findings were consistent with other reports that were carried out in Iran and were mentioned in the discussion section. For instance, in a study carried out in Isfahan province, self-immolation was more frequent among single, unemployed and young individuals [38]. Another study in South Khorasan province reported that self-immolation was more frequent among the married, those with elementary school education level, resident in urban areas, housewives and females [34]. In Ahvaz, the highest frequency of self-immolation was among the married and young people, females, housewives and illiterate individuals [39]. In addition, based on a study from Kermanshah province, the highest frequency of self-immolation was among females, young people, those with high school education degree, married individuals, unemployed and housewives [40], while in Lorestan province, self-immolation was more common in females, singles and those in the age group of 10–19 years [20]. According to a meta-analysis study conducted in Iran, self-immolation as a violent method was more prevalent in the western and southeastern provinces of Iran [41].

There are some differences between the outcomes of suicide based on the selection of a violent or non-violent suicide method. The comparison between our results (frequency of violent suicide methods) with those reported from Kermanshah [42], Shahrud [43] and Ilam [44] (frequency of nonviolent suicide methods) showed that the occurrence of successful suicide with non-violent methods were more common in men [42, 44], while in our study violent methods were more common in women. The lowest frequency of non-violent suicide was seen among illiterate individuals in the above-mentioned studies [42–44], but in our study, the lowest frequency of violent suicides was revealed among individuals with academic degree. The highest frequency of non-violent suicides was identified among students, followed by housewives in Kermanshah study [42], housewives followed by students in Shahrud study [43]; however, in our study, the highest frequency of violent suicides was seen in unemployed individuals. Based on other studies in Iran, non-violent suicides in both women and men were higher in urban regions [43, 44]; however, the present study revealed that violent suicides in men were more frequent in urban areas, but more frequent among women in rural areas. This discrepancy may be associated with socio-economic, customs and cultural difference as well as the accessibility to the means of suicide in different areas.

### Limitations

There were some limitations in this study; 1. The burns percentage and survival duration were not clear. 2. The exact details of suicide behaviors were unclear. 3- The exact reason of suicide was not detectable in completed suicides even though the family members or relatives who accompanied the victims completed the relevant questionnaires. 4. This study compared some variables in suicide attempts, but for the identification of risk factors associated with suicide; a case-control study was needed.

## Conclusion

This study revealed that more than 69% of suicides by physical methods were by self-immolation, which may be associated with easy access to flammable chemical substances, especially oil, that are commonly used domestically during winter, particularly in the rural areas. The difference in the suicide methods may result from the access rate to the instruments of suicide. On the other hand, the incidence rate of self-immolation was higher than the other physical methods, and is associated with severe psycho-somatic injuries on individuals, families and societies. Young women, being the active and requisite part of the societies, are more vulnerable to suicide. Thus, teaching life skills, health care techniques, social points, control and preventive programs are more needed than ever in order to reduce the incidence rate of violent suicide in families.
